# Behavioral Inhibition in the Second Year of Life Is Predicted by Prenatal Maternal Anxiety, Overprotective Parenting and Infant Temperament in Early Infancy

**DOI:** 10.3389/fpsyt.2022.844291

**Published:** 2022-06-03

**Authors:** Susanne Mudra, Ariane Göbel, Eva Möhler, Lydia Yao Stuhrmann, Michael Schulte-Markwort, Petra Arck, Kurt Hecher, Anke Diemert

**Affiliations:** ^1^Department of Child and Adolescent Psychiatry, Psychotherapy, and Psychosomatics, University Medical Center Hamburg-Eppendorf, Hamburg, Germany; ^2^Department of Child and Adolescent Psychiatry, Psychotherapy, and Psychosomatics, Saarland University Medical Center, Hamburg, Germany; ^3^Division of Experimental Feto-Maternal Medicine, Department of Obstetrics and Fetal Medicine, University Medical Center Hamburg-Eppendorf, Hamburg, Germany; ^4^Department of Obstetrics and Fetal Medicine, University Medical Center Hamburg-Eppendorf, Hamburg, Germany

**Keywords:** behavioral inhibition, internalizing personality traits, distress to novelty, early childhood, maternal overprotection, pregnancy-related anxiety

## Abstract

**Background:**

Behavioral inhibition, characterized by shyness, fear and avoidance of novel stimuli, has been linked with internalizing personality traits in childhood, adolescence and early adulthood, and particularly later social anxiety disorder. Little is known about the relevance of potential prenatal precursors and early predictors for the development of inhibited behavior, such as infant vulnerability and family risk factors like parental anxiety and overprotection. Pregnancy-related anxiety has been associated with both infant temperament and maternal overprotective parenting. Thus, the aim of this study was investigating the predictive relevance of prenatal pregnancy-related anxiety for behavioral inhibition in toddlerhood, by considering the mediating role of maternal overprotection and infant distress to novelty.

**Materials and Methods:**

As part of a longitudinal pregnancy cohort, behavioral inhibition at 24 months postpartum was assessed in *N* = 170 mother-child pairs. Maternal pregnancy-related anxiety was examined in the third trimester of pregnancy, and maternal overprotection and infant distress to novelty at 12 months postpartum.

**Results:**

Mediation analysis with two parallel mediators showed that the significant direct effect of pregnancy-related anxiety on child behavioral inhibition was fully mediated by infant distress to novelty *p* < 0.001 and maternal overprotection (*p* < 0.05). The included variables explained 26% of variance in behavioral inhibition. A subsequent explorative mediation analysis with serial mediators further showed a significant positive association between distress to novelty and maternal overprotective parenting (*p* < 0.05).

**Conclusion:**

Results indicate a predictive relevance of both infant and maternal factors for the development of behavioral inhibition in toddlerhood. Mothers who perceived more pregnancy-related anxiety showed more overprotective parenting and had infants with more distress to novelty. Further, mothers being more overprotective reported their child to be more inhibited in toddlerhood. Our findings also indicate the stability of reported infant distress to novelty as one aspect of later behavioral inhibition. Addressing specific forms of parental anxiety from pregnancy on and in interaction with child-related variables seems to be a promising approach for future studies and clinical interventions.

## Introduction

There is evidence of an interrelation of internalizing temperament and personality traits in the development from childhood to adolescence and adulthood (1, 2). Longitudinal studies indicate that specific temperament traits are not only evident in infants but also show stability within infancy and across childhood up into adolescence and adulthood (3–7). Still, research on temperament traits in younger children, their potential precursors and longitudinal outcomes from early on is limited. During recent years, the relevance of maternal mental health in the prenatal period for child outcomes has gained increased attention, and maternal distress has been linked not only to pregnancy and birth outcomes (8–10), but also to infant development and temperament (8, 11–15), such as “negative reactivity” (16). For internalizing characteristics across development, child behavioral inhibition (BI) in reaction to unfamiliar stimuli is one highly relevant risk factor for the development of socioemotional problems in later childhood or adolescence such as social anxiety (17–19). Moreover, the association between BI and later psychopathology seems to be related to infant and parental factors contributing to the maintenance of symptoms (20, 21). Due to the relevance of BI in the development of internalized psychopathology and for the refinement of potential starting points of interventions, it is important to identify predictive factors for BI, on parental as well as child side, from early on.

### Behavioral Inhibition

Behavioral inhibition (BI) is characterized by a tendency to react to unfamiliar stimuli (like persons, objects, situational contexts or tasks) as potential threats with avoidance, restraint or reluctance to approach (18, 22–24), often accompanied by staying close to the parent and freezing behavior (25). In novel social contexts, BI has been associated with less eye contact, smiling or vocalization (26).

Children with high BI might differ in how information processing systems and response systems interact with each other. Biased automatic and reflexive information processes might lead to an over-generalized perception of novel stimuli as potential threats. For children high in BI, a stronger stimulation of neural areas involved in automatic processing and fear responses, like the amygdala, the prefrontal cortex, and the striatum, have been identified (24, 27). Also, divergent activities in the dorsolateral prefrontal cortex have been reported, which are associated with more controlled information processing, like reward anticipation or impulse control (24). These dual information processing strategies in interaction with child BI might also explain developmental pathways from early to later inhibited behavior and internalizing characteristics, especially in social contexts.

Stability of reactivity to novel stimuli has been reported during infancy (18, 28), from infancy to the preschool age (29), and from childhood to early adulthood (4). Longitudinal studies indicate that especially for those children with stable and constantly high BI across assessment periods, less socially competent behavior with their peers (30) and higher rates of internalizing problems and anxiety disorders were reported from early childhood to the preschool age (31–34) and up into adolescence (35). Meta-analytic results highlight a predictive relevance of BI for anxiety disorders, particularly social anxiety disorder [SAD; (17, 19)]. Clauss and Blackford (17) reported a sevenfold increase in the risk of developing later SAD for children high in BI. Moreover, studies additionally indicate an association of BI at age 3 with juvenile and adult-onset depression (36, 37).

Regarding potential influencing factors on BI, previous studies reported mixed results on associations with maternal education (29, 38) and pregnancy complications (39, 40). Regarding the effect of child gender on the broader concept of temperament, mixed results have also been found. A meta-analysis by Else-Quest et al. (41) showed particularly effortful control to be more developed in girls, while gender differences in shyness or fear, favoring girls, were very small. Other studies reported inconsistent gender effects on BI, with girls exhibiting higher fear than boys, but not higher overall BI (18, 25), while some reported higher BI in girls than boys (29).

Nevertheless, little is known about the early development of inhibited behavior and its associated factors. Since divergent developmental trajectories have been identified and not all children with higher BI develop internalizing symptoms later on (21, 24, 34), it is thus important to understand the relevance of and the interplay between endogenous and exogenous factors contributing to the development of BI from pregnancy on.

### Association Between Maternal Pregnancy-Related Anxiety and Behavioral Inhibition

There is evidence for an association between children with BI having parents being more anxious and having more internalizing symptoms, anxiety disorders or depression (21, 42). Genetic heritability in the familial transmission of internalizing traits and symptoms has been identified as one important aetiological factor (21). Besides a sole genetic explanation, among other relevant pathways that have been discussed (43) the relation between genetic and environmental factors (GxE) has shown consistent relevance in the development of internalizing disorders (44). Thus, genes and environment are not independent but associated. For example, individuals at higher genetic risk are more likely to develop internalizing symptoms in reaction to environmental stressors (44, 45). Moreover, genes might be modified by the environment epigenetically (46). The model of fetal programming has gained increased attention during the last decades (9, 10, 47, 48). Specifically, maternal stress hormones produced by the maternal hypothalamus–adrenal–pituitary (HPA) axis are proposed to crucially influence the developing fetal HPA axis during sensitive periods of fetal brain development, resulting, for instance, in affected cerebral structures responsible for emotional and behavior regulation later on (16, 49). The specific underlying mechanisms are not clearly identified and are part of ongoing research (50).

Hence, a prenatal “programming” effect of prenatal anxiety on the regulation of reactivity to novelty during a critical period of development with lasting effects could play another central role in the transgenerational transmission of anxiety and inhibited behavior in “anxious families,” according to an interaction between genes and environment (10). Especially pregnancy-related anxiety (PrA) has been identified as a central variable for the peri- and postnatal outcomes in mother and child (10, 12, 16, 51, 52). PrA is defined as “nervousness and fear about the baby’s health, the mother’s health and appearance, experience with the health care system, social and financial issues in the context of pregnancy, childbirth, and parenting that are accompanied by excessive worry and somatic symptoms” [p. 121, (53)]. PrA has been found to be higher in women expecting their first child (54, 55), and has been linked to pregnancy complications, preterm birth and birth weight (56–59), as well as maternal postpartum mood (56, 60, 61).

Previous studies reported associations of PrA with child outcomes like attention regulation, motor development in infancy (62, 63), activity level (64, 65), mental development up to the age of 2 years (11, 66), higher fearfulness (64, 67, 68), as well as less optimal self-regulation in infancy and toddlerhood (14, 16). However, some studies could not confirm associations of PrA with infant reactivity up to 12 months of age, with equivocal results (14, 69).

Thus, based on these findings and the evidence of familial transmission of anxiety, the association between prenatal maternal PrA and BI in early childhood as a risk factor for later anxiety, in interplay with postnatal parental and child factors, needs further investigation.

### Maternal Overprotection and Its Potential Relevance for the Association Between Pregnancy-Related Anxiety and Behavioral Inhibition

One important exogenous environmental influence that has been identified to contribute to the longitudinal stability of BI and its later association with anxiety disorders is parenting behavior. Especially in the first years, the development of the infant’s ability to regulate states of arousal and distress is strongly related to parental co-regulation of the infant’s states, embedded in a dyadic, reciprocal interaction (70, 71). According to the mutual model by Gianino and Tronick (72), infant and parental risk as well as protective factors contribute to each other in a dynamic interplay. Particularly for children with any vulnerability such as high BI, sensitive parenting, supporting exploration and coregulation of infant distress in reaction to novelty might help to reduce an attention bias toward threat (73, 74). In contrast, parental overprotection (OP), controlling, critical, and also over-solicitous parenting has been associated with inhibited behavior in infants (21, 43, 75, 76) as well as with socially introverted or anxious behavior in preschool-aged children (77, 78). Further, parents of children showing high BI or fearfulness might more likely respond with more intrusive and controlling parenting (77) and less behavior supporting independence and exploration (79).

Overprotective and critical parenting as well as parental reaction to infant fear have been discussed as essential pathways determining the further development of infant anxiety, for example, by parental co-avoiding of unfamiliar stimuli (43). Lewis-Morrarty et al. (76) showed that maternal overcontrol at 7 years significantly predicted adolescent SAD and further had an interactive effect on the relation between higher BI in childhood and SAD in adolescence. Further, parental anxiety seems to be strongly associated with OP and avoidance (43). Pregnancy- and child-related anxiety has also been linked with more anxious parenting, such as maternal OP (55).

Murray et al. (80) proved that children with BI having mothers with SAD tended to be more avoidant to strangers. Vice versa, mothers with an anxiety disorder had more likely infants with higher reactivity and showed more avoidance behavior themselves (81). Relations between maternal OP and child internalizing characteristics might be especially strong in first-time mothers (82). There is further a greater probability for anxious mothers or fathers to perceive the child as more vulnerable (83–85). Accordingly, children of prenatally anxious and distressed parents are at higher risk of developing more “negative reactivity” in early infancy, which has been associated with later BI (16, 86).

Hence, the association between prenatal anxiety and child BI needs further investigation, considering the potentially mediating role of maternal OP on BI.

### Child Temperament and Its Potential Relevance for the Association Between Pregnancy-Related Anxiety and Behavioral Inhibition

A further relevant child factor associated with BI is child temperament, which comprises constitutionally based individual differences in reactivity and self-regulation (5). While dimensions of reactivity comprise negative mood, higher irritability, withdrawal in response to new stimuli, irregularity of biological rhythms, or “difficult” temperament early in infant development, the process of self-control develops and differentiates later on (87). BI as a subdimension of negative reactivity is characterized as more internalized form associated with shyness, withdrawal to novelty and a known risk factor for later (social) anxiety (16, 17).

According to the spectrum model, anxiety and BI might be on the same continuum, with BI representing a subclinical level of anxiety, sharing genetic covariation with temperamental traits such as withdrawal (21). In this context, the familial transmission of infant vulnerability described above is further confirmed by Stumper et al. (88), who showed that parental recalled own BI as a child mediated the relation between their child’s BI at preschool age and an anxiety disorder at the age of 9 years. Since there is evidence of the stability of temperamental traits during childhood (18), the relation of PrA to BI could be mediated by distress to novelty (DTN), a fearful temperament trait, in infancy. At the same time, as an endogenous factor, DTN itself might affect the environment, such as the parent-child-interaction, which in turn influences the development of potential child psychopathology (21, 43). Thus, the potentially mediating role of DTN on the association between PrA and BI needs further investigation.

### Aims of This Study

However, to the best of our knowledge, no study so far has investigated the association between prenatal anxiety and behavioral inhibition in toddlerhood by considering the role of infant temperament as well as maternal parenting, in terms of familial trajectories of anxiety from pregnancy on. Hence, the current study aimed to examine the predictive relevance of pregnancy-related anxiety for behavioral inhibition in early childhood. We expected prenatal pregnancy-related anxiety (PrA) to positively predict inhibited behavior (BI) up to 2 years of toddler’s age, and further expected this relation to be mediated by maternal overprotective parenting (OP) and infant distress to novelty (DTN) at 12 months postpartum: higher prenatal pregnancy-related anxiety would predict maternal overprotective parenting and higher infant distress at 12 months postpartum, which would both predict higher BI at 24 months postpartum. In addition, the potential association between the two mediators DTN and maternal OP was explored.

## Materials and Methods

### Study Design

The data for this longitudinal analysis were derived from a collaboration between two related ongoing population-based prospective studies based at the University Medical Center Hamburg-Eppendorf: The longitudinal pregnancy cohort PRINCE (“Prenatal Identification of Children’s Health”) initiated in 2011 is focusing on the feto-maternal immune cross talk [for more study details, see Bremer et al., (89)], as well as the PAULINE (“Prenatal Anxiety and Infant Early Emotional Development”) initiated in 2014 is investigating prenatal forms of anxiety and its association with infant emotional development and the mother-child relationship [for more study details, see Mudra et al., (55)].

### Study Sample and Procedure

Pregnant women were recruited upon initial presentation at the university after being sent by their resident gynecologists or by midwives between 2014 and 2017. Women aged 18 years or older, pregnant in their 12–14th gestational week with a singleton child were included in the study. Women with chronic infections, substance abuse, pregnancy after assisted reproductive technologies (ART) or lack of sufficient German language skills were excluded. Further, women with severe pregnancy complications, premature birth (<37 weeks gestation), low birth weight (<2,500 g) were excluded from the analysis since these factors may require intensive care for the child and substantial concern in the parent.

For the current analysis, questionnaire-based prenatal and postnatal data of the participants collected between the 34th and 36th week of pregnancy, as well as 12 and 24 months postpartum were included. Of the *N* = 181 women initially included in the study sample, *n* = 7 were subsequently excluded due to giving birth prematurely. Thus, the sample consisted of *N* = 174 participants. All participants signed informed consent forms. The study protocols were approved by the ethics committee of the Hamburg Chamber of Physicians (PV3694 and PV5574).

### Variables and Instruments

#### Behavioral Inhibition in Early Childhood

Behavioral inhibition in early childhood was assessed based on maternal report with the Retrospective Infant Behavioral Inhibition Scale [RIBI, (25)] when the child was 24 months old. The RIBI assesses child behavior within the last year on the three dimensions “distress to novelty,” “fear when confronted with activities or situations suggestive of minor risk” and “shyness,” which can be summed up in a total score of BI. The in total 20 items are rated on a 5-point scale, ranging from 0 = *Yes, likely* to 4 = *Not likely*. Higher sum scores indicate higher BI. Excellent internal consistency, construct and convergent validity of the RIBI were shown in previous studies (25). Scale reliability of the total BI sum score in the current sample was excellent (α = 0.90).

#### Pregnancy-Related Anxiety

Maternal PrA was assessed at 34 to 36 weeks of pregnancy with the German version of the Pregnancy-Related Anxiety Questionnaire, revised for all pregnant women regardless of parity [PRAQ-R2, (54); German version by Mudra et al. (90)]. The 10-item questionnaire focusses on current levels of fear of giving birth, worries about the child’s health and about own physical appearance. Items are rated on a 5-point scale ranging from 1 = *absolutely not relevant* to 5 = *very relevant*, with higher scores indicating higher anxiety levels. Construct validity and measurement invariance of the PRAQ-R2 were confirmed at different time points in pregnancy (54) and in different cultures (91). In this study, the PRAQ-R2 total score showed good scale reliability (α = 0.85).

#### Maternal Overprotection

Maternal OP at 7 months postpartum was assessed with the German self-report scale “Investigation of Maternal Attitudes for Mothers of Infants and Toddlers” [EMKK (92)], measuring maternal attitudes in taking care for their very young child. The subscale “overprotection out of fear” focusses on concerns regarding the child’s health (e.g., “I’m constantly concerned that something might happen to my child”) and own parenting (e.g., “Sometimes I am scared that I might hurt my child” Seventeen items are rated from 1 = *does apply* to 4 = *does not apply*. Higher scores indicate less problematic parenting behavior and thus less maternal OP. The EMKK has previously been used in samples of mothers with infants and toddlers (92–94). For the “overprotection out of fear” subscale, good scale reliability was previously reported [α = 0.80; (92)], which was confirmed in this study (α = 0.83).

#### Infant Temperament

Infant temperament regarding DTN was assessed based on maternal report when the child was 12 months old with a subscale of the German adaptation of the widely used Infant Behavior Questionnaire [IBQ, (95); German version by Pauli-Pott et al. (93)]. The 10-item subscale assessed infant’s distress and latency to approach novel or sudden stimuli like objects or new environments. The items are rated from 1 = *does apply* to 7 = *does not apply*, with higher scores indicating higher distress in the context of new stimuli. Scale stability and reliability across the first year postpartum were reported previously, with satisfying reliability for the DTN subscale [α = 0.79 at 10–12 months; (93)]. In this study, scale reliability was also satisfying (α = 0.74).

### Statistical Analysis

First, descriptive statistics and zero-order correlations of the included variables were calculated. To ease interpretation of results, the EMKK scores were then recoded for the correlation and mediation analyses, so that a higher score was indicative of higher problematic behavior.

To investigate the research question, a mediation analysis with two parallel mediators was performed. This model investigated the effect of the predictor PrA and the two mediators infant DTN and maternal OP on the outcome BI. Since DTN and maternal OP were both assessed at 12 months, a direction of effects cannot be investigated. However, studying their association within the mediation model was of further interest. Modeling was based on the assumption of potential, already prenatal influences of maternal anxiety on infant DTN, following the fetal programming hypothesis, and wide evidence of an individual effect of infant temperament on parenting behavior (21, 43). Thus, in a subsequent explorative analysis, a mediation analysis with serial mediators was conducted, to further investigate the association between DTN and maternal OP. Bivariate correlations and direct effects in the mediation model were considered significant at a level of *p* < 0.05. For the indirect effects, confidence intervals (CI) based on bias corrected bootstrap were investigated to evaluate significance. For the analyzed variables, missing values on the item level below 30% were replaced using the Expectation-Maximization algorithm. To address missing values on the case level, the mediation model in MPlus was estimated based on maximum likelihood estimation. Still, *n* = 4 cases were excluded from further analysis due to more than 50% missings in the data set. Thus, the subsequent analyses were based on *N* = 170 cases. The statistical analyses were conducted with IBM SPSS Statistics for Windows (Version 27) and MPlus, 8th edition (96).

## Results

### Sample Characteristics

Characteristics of the sample are listed in Table 1. Overall, the participants were well educated and had an average to high household income. Half of the sample had given birth to their first child and reported having a girl. Twenty percent reported pregnancy complications (including vaginal bleeding, pregnancy diabetes, high blood pressure, hyperemesis, premature contractions, cervical insufficiency or amniotic sac prolapse).

**TABLE 1:** Characteristics of the sample.

Maternal age at study intake (years), *M* (*SD*)	32.16 (3.67)
Range	22–44
**Educational level, *n* (%)**	
Up to middle school	41 (23.6)
High school graduation	45 (25.9)
University degree	85 (48.9)
No information provided	3 (1.7)
**Household income*^a^*, *n* (%)**	
≤1,000 €	5 (2.8)
1,001–2,000 €	13 (7.4)
2,001–4,000 €	64 (36.7)
≥4,001 €	79 (45.3)
No information provided	13 (7.5)
**Number of previous children, *n* (%)**	
0	86 (49.5)
1	73 (42.0)
2	12 (6.9)
No information provided	3 (1.7)
**Child gender assigned at birth, *n* (%)**	
Girl	88 (50.6)
Boy	85 (48.9)
No information provided	1 (0.6)
**Pregnancy complications, *n* (%)**	
Yes	34 (19.5)
No	139 (79.9)
no information provided	1 (0.6)

*^a^Household net income including child benefit.*

### Bivariate Correlations Among Included Variables

Associations between variables on a bivariate level are listed in Table 2, showing significant correlations in the expected directions: Higher BI at 24 months postpartum was positively associated with PrA, as well as with maternal OP and DTN at 12 months postpartum. Further, maternal OP and infant DTN were positively associated with PrA, respectively.

**TABLE 2:** Zero-order correlations for the outcome, predictor, and moderator variables.

	*M*	*SD*	BI (RIBI)	PrA (PRAQ-R2)	Maternal OP (EMKK*^a^*)	DTN (IBQ)
BI (RIBI)	28.79	12.35	–	0.224*	0.331**	0.471**
PrA (PRAQ-R2)	22.71	6.80		–	0.400**	0.235*
Maternal OP (EMKK*^a^*)	25.76	32.08			–	0.324**
DTN (IBQ)	31.26	0.48				–

BI, behavioral inhibition; RIBI, Retrospective Infant Behavioral Inhibition Scale; PrA, pregnancy-related anxiety; PRAQ-R2, Pregnancy-Related Anxiety Questionnaire-revised for all pregnant women regardless of parity; OP, overprotection; EMKK, investigation of maternal attitudes for mothers of infants and toddlers; DTN, distress to novelty; IBQ, Infant Behavior Questionnaire. ^a^For EMKK, scores were inversed, so that a higher score indicates higher maternal OP. *p < 0.05; **p < 0.001.

### Mediation Analysis

**FIGURE 1:**
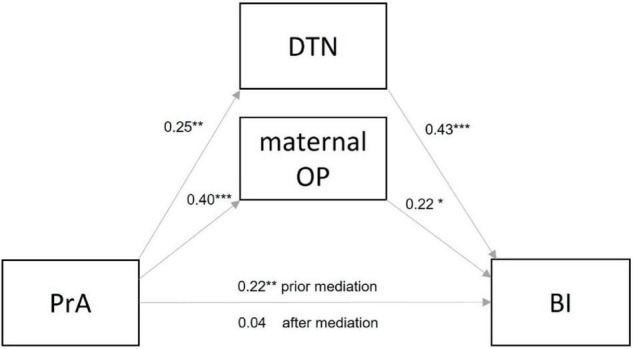
Standardized estimates for the mediation model between pregnancy-related anxiety (PrA) and infant behavioral inhibition (BI), with the two parallel mediators infant distress to novelty (DTN) and maternal overprotection (OP). ***p < 0.001; **p < 0.01; *p < 0.05.

First, a direct effect of PrA on BI was investigated, which was found to be significant (β = 0.22, *z* = 2.96, *p* < 0.01), indicating that higher PrA predicted higher BI at 24 months. However, after adding the mediators to the model, this effect was no longer significant (β = 0.04, *z* = 0.53, *p* = 0.597). Both mediators maternal OP (β = 0.22, *z* = 2.50, *p* < 0.05) and DTN (β = 0.43, *z* = 5.37, *p* < 0.001) had significant effects on BI, with higher maternal OP and higher DTN at 12 months predicting higher BI at 24 months postpartum. Further, significant positive effects of PrA on both maternal OP (β = 0.40, *z* = 5.06, *p* < 0.001) and infant DTN (β = 0.25, *z* = 3.32, *p* < 0.01) were found. Thus, higher PrA predicted higher maternal OP and infant DTN at 12 months postpartum. The total indirect effect was also found to be significant (β = 2.31, *z* = 3.21, 95% CI [1.82, 5.62]), and the specific indirect effects of PrA on BI were significant for both paths, with maternal OP (β = 0.9, *z* = 2.27, [0.45, 3.21]) and infant DTN (β = 0.11, *z* = 2.70, [0.67, 3.70]) as mediators. Thus, the association between PrA and BI was in this model fully mediated by maternal OP and infant DTN: higher PrA predicted higher maternal OP and higher DTN at 12 months postpartum, with both mediators predicting higher BI at 24 months postpartum (see Figure 1 for final model). Overall, 26% (*R*^2^_*adj*_ = 0.25) of the variance in BI were explained by the included variables.

### Explorative Analysis

The association between maternal OP and DTN was further investigated in a serial two mediator model (see Supplementary Figure 1). DTN was considered as the first mediator in the sequential model, followed by maternal OP. Thus, an additional path from DTN to maternal OP was investigated, as well as an indirect effect of PrA on BI considering DTN and maternal OP in sequence. In this model, the results of the main model were confirmed. Additionally, a significant effect of DTN to maternal OP was confirmed (β = 0.24, *z* = 2.59, *p* < 0.05). Further, a specific indirect effect of PrA on BI with DTN and maternal OP was confirmed (β = 0.01, *z* = 1.47, 95% CI [0.00, 0.49]).

### Sensitivity Analysis

The significant effects in the main and explorative analysis remained significant after controlling for potentially influencing factors on infant BI, and no significant effects of maternal education (β = −0.02, *z* = −0.16, *p* = 0.873), parity (β = −0.03, *z* = −0.38, *p* = 0.702), pregnancy complications (β = 0.06, *z* = 0.59, *p* = 0.551), or child gender (β = −0.002, *z* = −0.03, *p* = 0.978) on infant BI were found.

## Discussion

This study investigated the effect of PrA in the third trimester of pregnancy on BI in early childhood, considering a mediating effect of maternal OP and child DTN at 12 months postpartum. In this analysis, the initial predictive effect of PrA on BI was fully mediated by maternal OP and child DTN at 12 months postpartum. Thus, more anxious mothers during pregnancy did not only report higher levels of overprotective parenting but also perceive their child as showing higher distress to novel stimuli 1 year after birth. Further, maternal parenting and infant temperament significantly predicted BI at 24 months postpartum. Moreover, a subsequent explorative analysis of the mediators showed that DTN was positively associated with maternal OP at 12 months postpartum. Our study provides a valuable insight into the early interplay of infant and maternal factors as potential endogenous and exogenous predictors of BI, already including prenatal influences.

Regarding maternal prenatal anxiety, the current results on a significant relation between PrA in the third trimester of pregnancy and infant temperament in infants 12 months of age are in line with previous studies identifying PrA as an independent predictor of infant as well as early childhood temperament (62, 68, 86, 97). Following the hypothesis of fetal programming, the effect of high levels of anxiety might further depend on time periods in which fetal neural development is specifically vulnerable to influences by maternal stress hormones. Previous results indicate an influence of PrA at different time periods during pregnancy. However, Blair et al. (97) identified only PrA in the first trimester as being related to child negative affectivity at the age of 2 years, even after controlling for postpartum anxiety. Similarly, Huizink et al. (62) found an association between PrA in early pregnancy and infant attention regulation 3 and 8 months after birth, while McMahon et al. (69) could not prove an effect of PrA in the third trimester on infant temperament at 4 months postpartum. Contrarily, Thiel et al. (86) reported general anxiety and particularly fear of childbirth at 32 weeks of pregnancy being associated with difficult infant temperament 8 weeks after birth.

Even though PrA dimensions differ in their developmental course and intensity across pregnancy (55), and PrA has been investigated at different stages of pregnancy during the past decades, no clear pattern of vulnerable periods during pregnancy for a stronger programming effect of prenatal anxiety on infant reactivity has been identified yet (14, 16).

Most investigations of prenatal anxiety and infant temperament focused on infants in the first year of life (16). Among the studies on children of 18 months and older, mixed results have been reported, depending on the form of prenatal stress and the age of the investigated children. While some studies reported no effect of prenatal stress on child temperament or behavior at 3, 12, 18, or 24 months (98, 99), others reported associations of prenatal stress with less regularity, persistence and attention span at 16–18 months, and more behavioral problems in children 2 years of age (100, 101). Further, Blair et al. (97) found an association between particularly higher PrA and negative affectivity at 2 years postpartum, but no effect for peripartum general anxiety. Also, the current study showed a significant direct effect of PrA on BI at 24 months, which, however, turned out to be fully mediated by DTN and maternal OP. Since Blair et al. (97) and others did not assess infant temperament as a mediator earlier than 24 months or BI in particular, the results are not fully comparable. Hence, our findings highlight the significant influence of PrA in pregnancy on BI in early childhood via DTN in infancy, supporting the hypothesis of a potential programming effect of prenatal anxiety on infant distress to novel stimuli and behavioral inhibition. Results on epigenetic mechanisms in the transition of prenatal anxiety on child behavior are limited thus far (102). Nevertheless, to replicate the current findings and differentiate underlying effects in more detail, future longitudinal study designs should include recalled information on BI in both parents, combined with genetical, biological and behavioral data, starting early in pregnancy or even preconceptionally (10, 21, 43, 103).

In the current analysis, the child’s DTN at 12 months further predicted BI at 24 months with the strongest association among the investigated variables. The effect also indicates a stability of child temperament traits and confirms DTN as a precursor of BI. This finding is in line with previous studies showing an association between infant reactivity at 4 months of age with BI in infants at 14 months in laboratory assessment (73). Hence, assessing early child temperament during regular pediatric examinations might help identify infants at higher risk for BI from early on, for example, using parental questionnaires as a feasible method. While several studies reported a stability of temperament across infancy or between childhood and adolescence assessed by observations as well as questionnaires (6, 7), our results may offer an additional insight into perinatal and infant predictors of BI as known risk factors for internalizing traits. Moreover, in the interpretation of data there is a substantial development between infant age of 12 and 24 months to consider regarding the child’s activity level, autonomy granting as well as the perception of external parental control (104).

Thus, instead of a linear developmental process of temperament traits, there is evidence of a complex interplay between central endogenous internalizing infant traits and relevant environmental exogenous influences such as the parent-infant-interaction, which have rarely been investigated in the same study design (21). Hence, the current study further identified postpartum maternal overprotective parenting out of fear at 12 months as the second significant mediator for the relation between prenatal PrA and child BI at 24 months. Previous studies and own analyses give evidence for a relation between PrA and maternal OP at 7 months postpartum (BR90a),. Higher levels of PrA have been found to be significantly associated with more overall and child-related dissatisfaction with motherhood 3 weeks after birth and more parenting distress at 3 months postpartum (106, 107). The differentiation of specific forms of parental anxiety in the context of parenting should be another relevant aspect to consider. Pregnancy- or child-related anxiety with a stronger relational focus than general anxiety might affect parenting and the experience of child and parenthood, in particular. For example, Stuhrmann et al. (61) found that women with higher prenatal PrA showed more signs of depression and less self confidence in caretaking postpartum, which could promote an anxious, rather overprotective parenting style later on.

Maternal OP has also been also independently associated with child BI (21, 108). A meta-analysis by Van der Bruggen et al. (109) showed that independent of postpartum maternal anxiety, maternal controlling parenting behavior was related to the development of child anxiety. This further highlights the central mediating role of overprotective parenting for the influence of prenatal anxiety on the development of child BI in the current study. Parents who experience more PrA prenatally seem to be more overprotective in caretaking, which in turn promotes more inhibited behavior in their children. The developmental increase of activity level and autonomy granting between 12 and 24 months in the infants of the current study may have further provoked more OP, particularly in (prenatally) anxious parents (43, 85). In the current investigation maternal avoiding behavior out of fear has not been assessed which could be a beneficial addition in future studies (57, 80). Moreover, parents’ own BI in relation to the development of PrA and different parenting styles could be considered, according to Stumper et al. (88), to differentiate the role of PrA in the complex development of internalizing traits even more precisely.

Taken together, and as described by Lebowitz et al. (43) several pathways exist in the transmission of familial anxiety. Besides a higher genetical or fetal vulnerability to develop a fearful temperament in infants of prenatally anxious mothers, infant DTN might then again influence maternal parenting (5, 110). As the current study shows, the perception of the child as being more reactive, can affect parenting. Results of our explorative analysis indicate that perceiving the child as being more distressed to novelty might a reinforce parental tendency to overprotect parenting. Although infant gender showed no significant effect in our sensitivity analysis, an effect of gender on infant temperament cannot be ruled out (18, 25, 29, 41) and might also affect parenting behavior. For example, Hughes et al. (111) showed that while maternal autonomy support in parenting was not associated with child temperament nor with child gender at the age of 14 months, fathers displayed less autonomy support with their son than fathers with their daughter. However, Verhoeven et al. (112) could not find gender-related effects of paternal or maternal parenting on elementary school-aged children or adolescents, but specific effects of paternal or maternal over control or autonomy granting on their child’s anxiety in childhood and adolescence. These results and further literature on differences in parenting behavior in anxious mothers and fathers (113–115) highlight the importance of investigating the individual and interactive effects of both parents’ behavior on child development, which should be considered in future research. Additionally, studies assessing the parent-child interaction over several assessment points could further shed light on the interplay between infant temperament and early parenting and its effect on later child development (21, 116).

### Limitations

The main strength of the current study is the longitudinal investigation of the association between PrA and BI in early childhood by considering endogenous and exogenous factors, namely infant vulnerability, in terms of early temperament, as well as maternal parenting, in terms of overprotection. Nevertheless, the current study also has several limitations. First of all, a more complex analysis of the interplay between endogenous and exogenous factors was limited by the sample size and should be replicated in a larger sample. Due to the rather homogenous socioeconomic characteristics of participants and the exclusion of preterm birth, the current study might further have missed families at higher psychosocial risk and higher levels of prenatal anxiety or postpartum overprotection. Moreover, the paternal perspective with its potentially differing parenting style and perception of the child was missing and should be included in future investigations. Furthermore, an assessment of child temperament based on maternal reports only might lead to potential bias, which should be reduced by an additional assessment using direct child observations in future studies. Hence, a multi-informant approach, including direct observations of the child and multiple perspectives from both parents, strangers, and teachers, might broaden the picture of the child and enhance the quality in future assessments of fearful temperament or inhibited behavior (117–120).

## Conclusion

The current study provides findings of scientific and clinical relevance giving further evidence for the transmission of internalizing traits in an interplay between parental and infant factors from early on. Our results show an effect of prenatal maternal pregnancy-related anxiety on child behavioral inhibition at 24 months of age as a risk factor for later child anxiety, crucially mediated by infant early temperament as well as maternal overprotective parenting. Identifying highly anxious parents already prenatally can be beneficial for refining professional support according to the needs of affected parents and their children.

## Data Availability Statement

The datasets presented in this article are not readily available because of the ethical committee’s decision. Requests to access the datasets should be directed to SM, s.mudra@uke.de.

## Ethics Statement

The studies involving human participants were reviewed and approved by the Ethics Committee of the Hamburg Chamber of Physicians (PV3694 and PV5574). The participants provided their written informed consent to participate in this study.

## Author Contributions

SM developed the question under research, drafted and revised the manuscript, contributed to investigation, and conceptualization of the research design. AG conducted the statistical analyses, drafted and revised the manuscript, contributed to investigation, and development of the research question. EM contributed to the development of the research question and revision of the manuscript. LS contributed to investigation, development of the research question, and revision of the manuscript. MS-M, PA, and KH contributed to conceptualization of the research design and revision of the manuscript. AD contributed to investigation, conceptualization of the research design, and revision of the manuscript. All authors contributed substantially to this work and approved the final version of this manuscript.

## Conflict of Interest

The authors declare that the research was conducted in the absence of any commercial or financial relationships that could be construed as a potential conflict of interest.

## Publisher’s Note

All claims expressed in this article are solely those of the authors and do not necessarily represent those of their affiliated organizations, or those of the publisher, the editors and the reviewers. Any product that may be evaluated in this article, or claim that may be made by its manufacturer, is not guaranteed or endorsed by the publisher.

## Abbreviations

BI: behavioral inhibition
DTN: distress to novelty
OP: overprotection
PrA: pregnancy-related anxiety
SAD: social anxiety disorder.
