# Supplementary figures and images for: Behavioral Inhibition in the Second Year of Life Is Predicted by Prenatal Maternal Anxiety, Overprotective Parenting and Infant Temperament in Early Infancy
Source: Front Psychiatry. 2022 Jun 3;13:844291. doi: 10.3389/fpsyt.2022.844291 (PMC9203734; doi:10.3389/fpsyt.2022.844291)

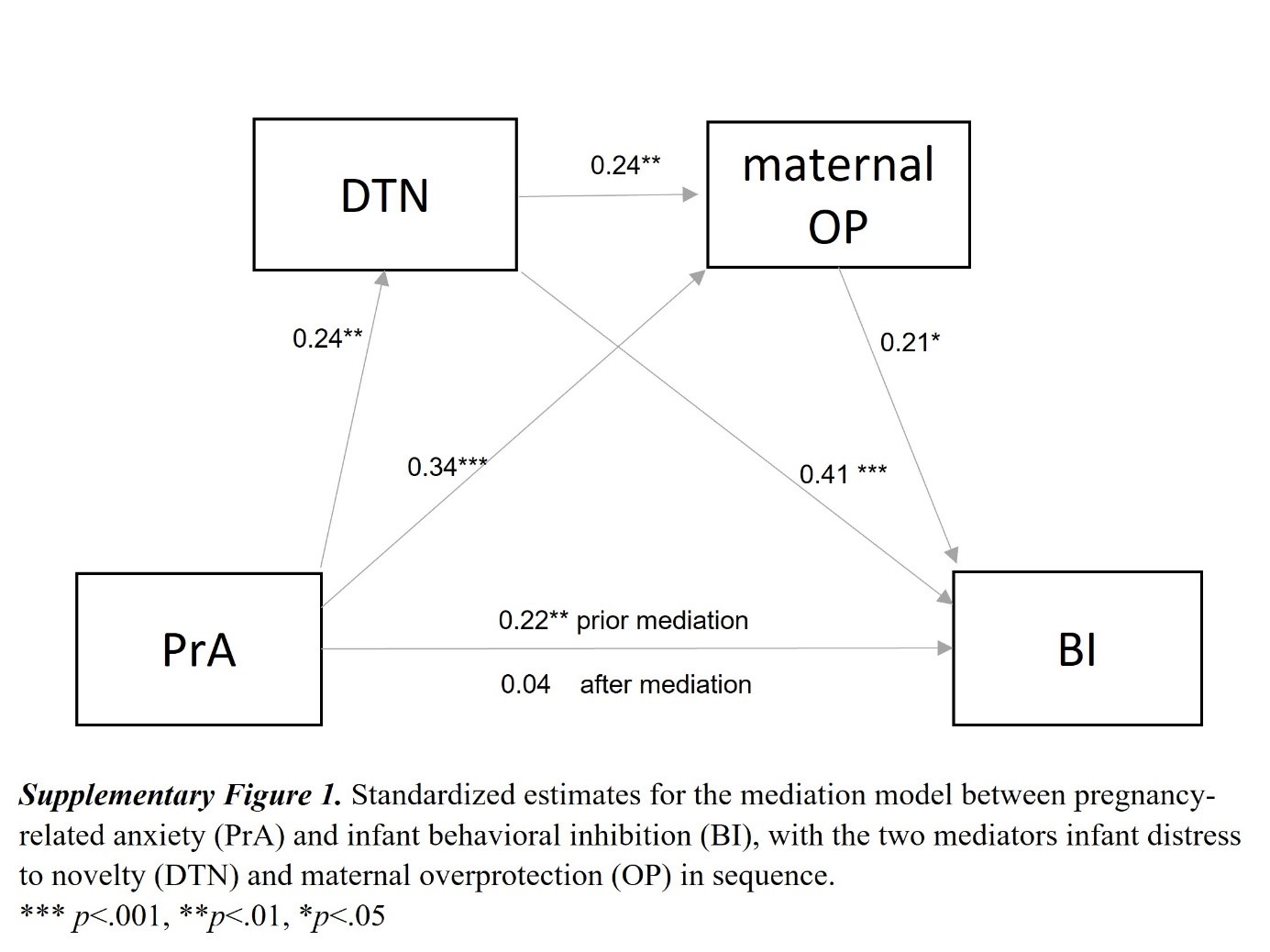

Supplement: Supplementary file 1 [file Image_1.jpg]
